# UWB Frequency-Selective Surface Absorber Based on Graphene Featuring Wide-Angle Stability

**DOI:** 10.3390/s23052677

**Published:** 2023-03-01

**Authors:** Zhefei Wang, Jiajun Huang, Dongjiao Sun, Qingsheng Zeng, Mingxin Song, Tayeb A. Denidni

**Affiliations:** 1 School of Electronic and Information Engineering, Nanjing University of Information Science and Technology, Nanjing 210044, China; 2 College of Astronautics, Nanjing University of Aeronautics and Astronautics, Nanjing 210016, China; 3 College of Applied Technology, Hainan University, Danzhou 571737, China; 4 Institut National de la Recherche Scientifique, Université du Quebec, Montreal, QC H5A 1K6, Canada

**Keywords:** absorber, ultra-wideband, frequency-selective surface, angular stability, graphene

## Abstract

In this paper, an ultra-wideband and polarization-insensitive frequency-selective surface absorber is presented with oblique incident stable behavior. Different from conventional absorbers, the absorption behavior is much less deteriorated with the increase in the incidence angle. Two hybrid resonators, which are realized by symmetrical graphene patterns, are employed to obtain the desired broadband and polarization-insensitive absorption performance. The optimal impedance-matching behavior is designed at the oblique incidence of electromagnetic waves, and an equivalent circuit model is used to analyze and facilitate the mechanism of the proposed absorber. The results indicate that the absorber can maintain a stable absorption performance with a fractional bandwidth (FWB) of 136.4% up to 40°. With these performances, the proposed UWB absorber could be more competitive in aerospace applications.

## 1. Introduction

A frequency-selective surface (FSS), also known as an artificial electromagnetic metasurface, is a kind of periodic electromagnetic modulation structure [1,2]. Because of its excellent electromagnetic wave modulation ability, it has attracted scholars’ attention in recent years [3,4]. Compared with traditional materials, designs made of FSS have more obvious advantages such as thinness, a wider bandwidth, and higher efficiency in most cases. Therefore, FSS is more competitive in the aerospace, antenna, microwave, optical instruments, and other fields. Among them, the FSS absorber plays a critical role in the field of stealth technology. In the stealth field, a conventional term to quantify radar detectability is known as radar cross-section (RCS), which is defined as the equivalent area of a target as seen by the radar [5]. Reducing the RCS of the target is the most effective way to enhance the stealth effect. Some techniques have been proposed to reduce the RCS to better achieve the purpose of stealth, such as applying radar-absorbing materials [6], shaping the geometry [7], employing polarization conversion metasurfaces [8], and so on. Among these, frequency-selective absorbers have been extensively studied in the stealth field due to their characteristics and advantages in recent years.

In the available literature, most of the FSS absorbers are composed of multilayer structures and their loss modules are usually resistors, resistive inks, or resistive sheets. These absorbers can be categorized as single-band or multiband according to the resonance of the structures [9,10]. The absorption bandwidth can be enhanced by using a multiple resonant structure [11], multiple-layer [12], resistance [13], and fractal patterns [14]. To meet the polarization insensitivity requirement, symmetrical structures have been adopted [15]. According to the published research, a good loss module is graphene, which is a 2D carbon material with exceptional flexibility and outstanding conductivity [16]. Using graphene flake instead of pure resistive films can reduce the insertion loss and be fabricated together with metal patterns [17]. An ultra-wideband FSS absorber using hybrid metal–graphene structure with good angular stability has been presented [18]. Up until now, many FSS-based absorbers with lumped elements have been proposed as circuit analog absorbers, which focus on angular stability [19], broadband [20,21,22], ultrathin [23], dual-band [24,25], tunable [26,27], switchable [28], low-profile [29], transparency [30], polarization insensitivity [31], and so on. Furthermore, a 3D absorber with a relatively small volume was proposed, which effectively avoided the appearance of grating lobes and has good angular stability [32]. The oblique angle sensitivity problem of conventional Jerusalem cross FSS was resolved by enhancing the capacitance loading, especially for TM polarization [33]. A novel miniaturized concept named strong-coupled frequency selective surface with excellent angular stability has also been presented [34]. A square ring with concave–convex deformation was designed, which expanded the current path to realize the miniaturization of the absorber, which decreased the influence of the oblique incident on the absorption performance [35]. However, angular stability is still a crucial problem to be further improved by scholars.

To solve this problem, designing the optimal impedance matching effect at the oblique incident angle is proposed in this paper. Furthermore, using graphene coating can also obtain better broadband absorption and angular stability on this basis. The results of the research show that the performance of the absorber designed by this method will not deteriorate with the change in the incident angle within the range of 0–40°. On the contrary, its absorption band coverage is improved. The proposed ultra-wideband absorber with angular stability and polarization insensitivity may have a high application potential in the field of stealth.

## 2. Materials and Methods

**Figure 1:**
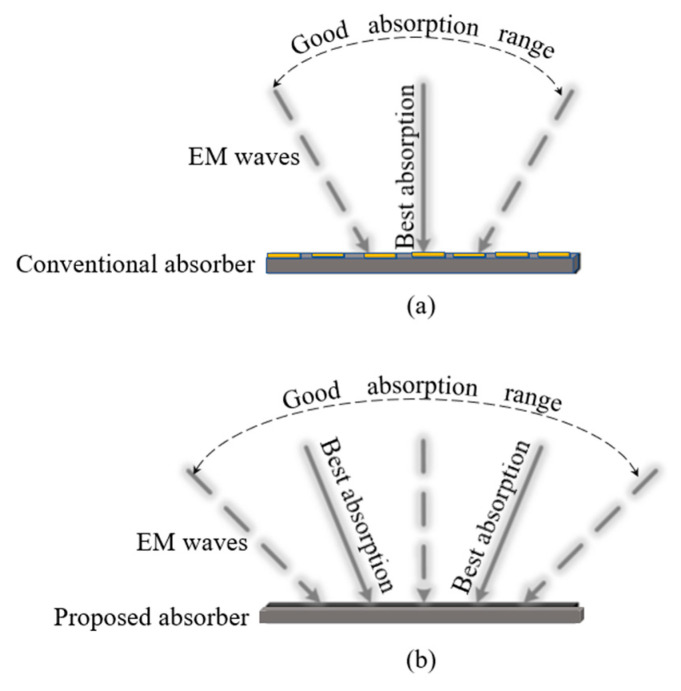
Difference between the conventional structures and the proposed structure. The best absorption incident angle and good absorption range of (**a**) conventional absorbers and (**b**) the proposed absorber.

So far, there has been a common problem with conventional absorbers that the absorption performance worsens with the increase in the incident angle. As shown in Figure 1a, conventional absorbers have the best absorption performance under the normal incident angle. When the incident angle increases, the absorption performance deteriorates. As can be seen from Figure 1b, the new method proposed to solve this problem had its best absorption performance designed to be at the oblique incidence. As a result, the proposed absorber had a larger good absorption range than conventional absorbers.

The absorption mechanism of the absorber is the impedance matching principle. For the presented structure, the input impedance of the absorber was close to 377 Ω in a wide band, and the imaginary part was close to zero under oblique incidences, which matched the wave impedance of the air conjugate. At this time, the electromagnetic energy irradiated in the absorber hardly reflected and most entered the absorber. Graphene was used as the lossy material, so the electromagnetic energy entering the absorber was quickly lost to achieve perfect absorption.

**Figure 2:**
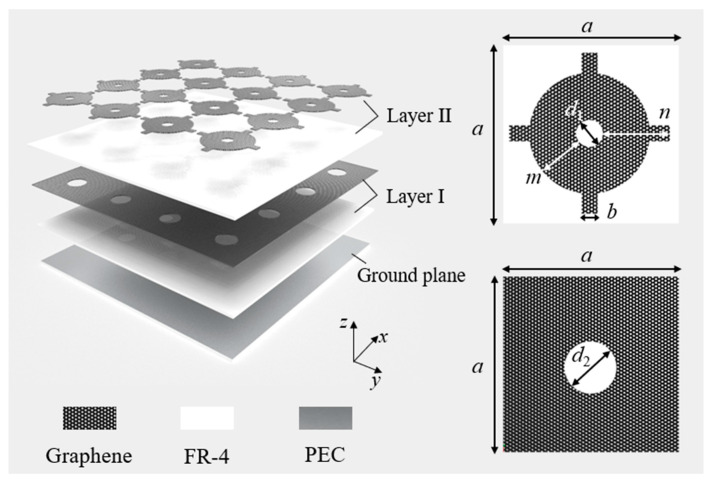
The unit cell structure of the proposed absorber.

The unit cell geometry of the proposed FSS absorber is shown in Figure 2. The graphene-based lossy FSS was imprinted on a 0.25 mm thick low-cost FR-4 substrate (*ε* = 4.3). The purpose of using a two-layer lossy structure was to obtain a large bandwidth. Both the lossy layer I and lossy layer II had an absorption band. Combining the two lossy layers obtained an ultrawide absorption band. The symmetric graphene coating structure made the absorber insensitive to different incident polarization. To ensure zero transmission, a ground plane was provided on the bottom of another FR-4 substrate of the same thickness. After calculation and simulation, the optimized geometrical design parameters are shown in Table 1. Among them, *h*_1_ represents from the lower part of the bottom plate to the lower surface of lossy layer I, and *h*_2_ represents from the lower surface of lossy layer I to the lower surface of lossy layer II.

The graphene was composed of graphene flakes, carbon black, solvents, related binders, and dispersants. To ensure the carbon black and graphene flakes were uniformly dispersed, they were mixed and ultrasonically dispersed in a planetary mixer/deaerator (MAZERUSTAR KK300SSE made by KURABO in Osaka, Japan) and an ultrasonic cleaner. Then, the graphene ink was printed on the prepared FR-4 substrate and cured at a high temperature [36]. The complex dielectric function of the graphene was evaluated using the conductivity of graphene, where the conductivity of graphene is dependent upon the chemical potential. The surface conductivity of graphene was obtained from Kubo’s formula [37]:

(1) $$\sigma_{g}=\frac{je^{2}}{4\pi \hbar^{2}}\ln\left[\frac{2\left|\mu_{c}\right|-\left(\omega +j/\tau \right)\hbar }{2\left|\mu_{c}\right|+\left(\omega +j/\tau \right)\hbar }\right]+\frac{je^{2}k_{B}T}{\pi \hbar^{2}\left(\omega +j\tau^{-1}\right)}\left[\frac{\mu_{c}}{k_{B}T}+\ln\left(1+e^{-\frac{\mu_{c}}{k_{B}T}}\right)\right]$$

where e is the electron charge, ω is the angular frequency, $k_{B}$ is the Boltzmann’s constant, ℏ is the reduced Planck’s constant, and *T* is the temperature fixed to 300 K.

Then, the value of the permittivity of graphene was calculated by using the formula [38]:

(2) $$\varepsilon =1+\frac{i\sigma_{g}}{\omega \varepsilon_{0}d_{g}}$$

where *d_g_* is the thickness of the graphene layer. The permittivity of the graphene was 2.3 on the research frequencies [39].

**Figure 3:**
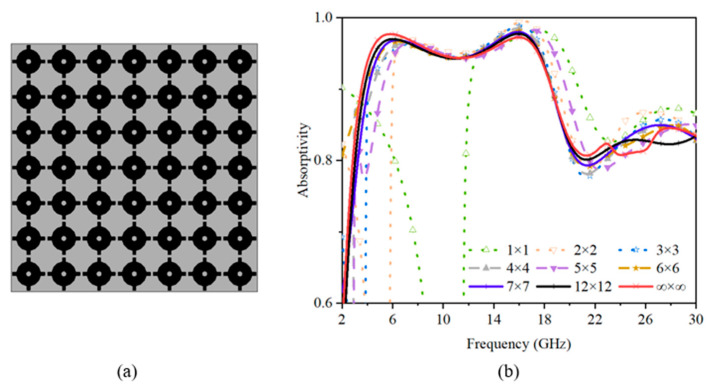
The finite-size final structure. (**a**) The finite-period structure (7 × 7). (**b**) The absorptivity of the structure with different periods.

To further facilitate this research, the absorption performances of different finite-size structures were modeled and simulated through the FSS FULL STRUCTURE function of CST Microwave Studio, as presented in Figure 3. It can be seen that, when the unit cells were less than 6 × 6, the absorption performance of the proposed structure at lower frequencies was not very good due to the relatively large wavelength, and it became better as the number of units increased. With the unit of 7 × 7, the absorption behavior was similar to the one with an infinite periodic structure. Therefore, to obtain more accurate results, the unit cell of the final structure should be more than 7 × 7.

**Figure 4:**
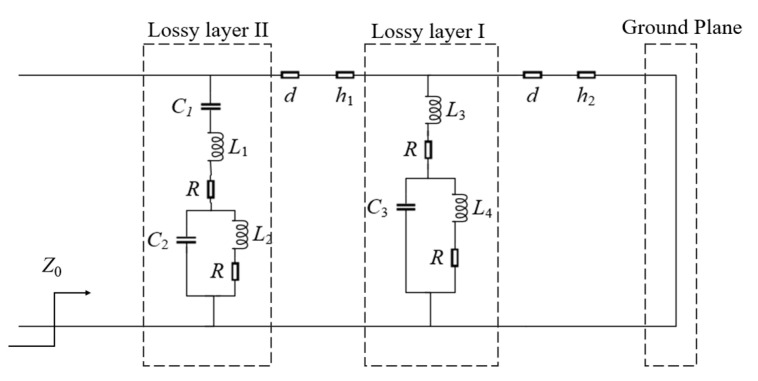
Equivalent circuit model of the proposed absorber.

As shown in Figure 4, the functioning of the proposed structure was further analyzed based on the corresponding equivalent circuit model (ECM). The incident wave leads to the generation of surface currents. Due to these surface currents on metallic designs, inductive and capacitive effects are induced, which can be modeled by the corresponding inductors and capacitors, respectively. The dielectric and air layers can be replaced by separate transmission lines. The thickness of the lossy layer I and the lossy layer II can be characterized using a short transmission line model with characteristic impedance $Z_{d}=\;Z_{0}/\sqrt{\varepsilon_{t}}$ and length equivalent to the thickness of substrates (*d*). Here, *Z*_0_ = 377 Ω represents the free space wave impedance, and $\varepsilon_{t}$ is the relative permittivity of the dielectric material.

To better predict the frequency response of the proposed FSS by ECM, the capacitors (*C*_1_, *C*_2_, and *C*_3_) and inductors (*L*_1_, *L*_2_, *L*_3_, and *L*_4_) in Figure 3 above were approximately calculated by the equations based on [40,41,42] as follows:

(3) $$L=\mu_{{}_{0}}\mu_{t}\frac{a}{2\pi }\mathrm{In}[\frac{1}{\sin(l\pi /2a)}]$$

(4) $$C=\varepsilon_{0}\varepsilon_{t}\frac{2a}{\pi }\mathrm{In}[\frac{1}{\sin(d\pi /2a)}]$$

where $\mu_{0}$ and $\varepsilon_{0}$ are the vacuum permeability and vacuum dielectric constant, respectively, *a* is the period of the unit structure, and *l* and *d* are the length and width of the graphene structure, respectively.

The impedance of the lossy layers I and II can be expressed by the following formula, respectively.

(5) $$Z_{1}=\frac{1}{j\omega C_{1}}+j\omega L_{1}+R+\frac{\frac{1}{j\omega C_{2}}(j\omega L_{2}+R)}{\frac{1}{j\omega C_{2}}+j\omega L_{2}+R}$$

(6) $$Z_{2}=j\omega L_{3}+R+\frac{\frac{1}{j\omega C_{3}}(j\omega L_{4}+R)}{\frac{1}{j\omega C_{3}}+j\omega L_{4}+R}(1).$$

After fitting and optimizing, the final values of the circuit parameters were obtained with the values shown in Table 2.

In this work, the optimal impedance matching effect was designed at the incident angle of 45°. According to Formulas (3) and (4), it can be seen that the increasing incident angle mainly influenced the value of d and the period along the *x* axis when the incidence angle increased along the +*x* axis. Theoretically, the changes in C and L can be estimated by substituting $a/\sqrt{2}$ and $d/\sqrt{2}$ into Formulas (3) and (4) to obtain more accurate values at 45°. The final values of the capacitances and inductances vary within a small range, which requires further slight adjustments. Finally, the increasing incident angle mainly influenced the values of capacitances, which changed to following results: *C*_1_ = 0.171 pF, *C*_2_ = 0.0318 pF, and *C*_3_ = 1.5 pF.

## 3. Results and Discussion

### 3.1. Results of the Equivalent Circuit Model

**Figure 5:**
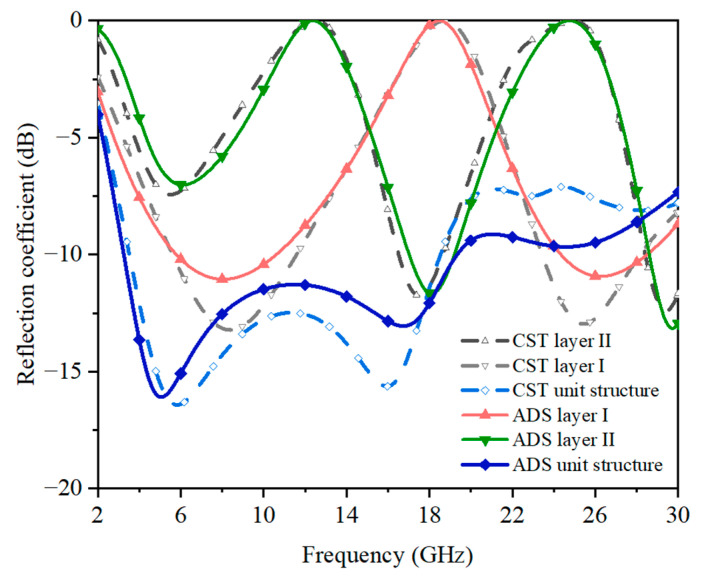
The ECM results of the ADS and the full-wave results of the CST.

The ECM and unit cell geometry of the proposed FSS absorber were simulated using Keysight Advanced Design System (ADS) software and CST Studio Suite (CST) software, respectively. The simulated reflection coefficients (S_11_) of the lossy layers are shown in Figure 5; under the normal incident angle, the reflection band below −10 dB ranged from about 3 GHz to 19 GHz. Both the circuit simulation and the full-wave simulation showed close matches with each other. Furthermore, the proposed FSS absorber geometry had axial symmetry and, therefore, exhibited a polarization-insensitive characteristic.

**Figure 6:**
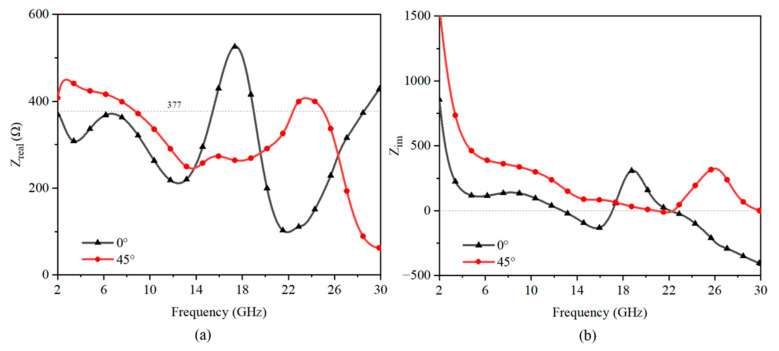
The impedance of the presented absorber at 0° and 45°. (**a**) The real part and (**b**) the imaginary part of the impedance.

**Figure 7:**
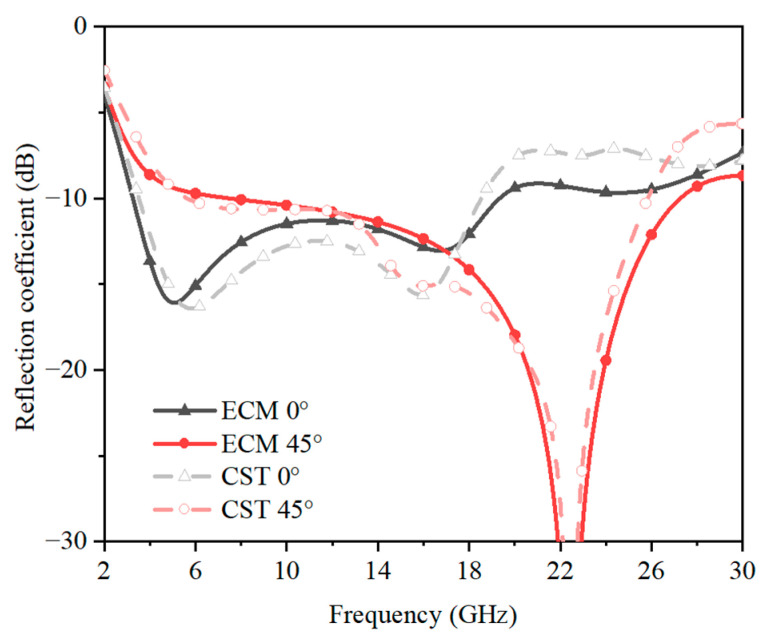
The reflection coefficient at 0° and 45° of the ECM and CST.

Moreover, the input impedance of the absorber was extracted through the simulated S parameter, to quantitatively explain the working mechanism of the absorber. The real and imaginary parts of the impedance at 0° and 45° are shown in Figure 6, which proves that the impedance at 45° was indeed better than that at 0°. As shown in Figure 6a, the real part of the impedance of the absorber at 45° was closer to 377 Ω than that at 0° from about 4 GHz to 26 GHz. At around 6 GHz and 15 GHz at 0° and around 22 GHz at 45°, the real part was very close to 377 Ω, and the imaginary part was close to 0. As a result, the impedance matching should be better. This corresponded to the absorption peaks in Figure 7. The frequency points close to zero of the imaginary part and those close to 377 of the real part both moved to high-frequency bands, which means that the absorption bandwidth may become larger. This corresponded to the result in Figure 7, which showed clearly that the absorption band extended from 18 GHz to 26 GHz.

### 3.2. Oblique Incidence Absorption Performance

**Figure 8:**
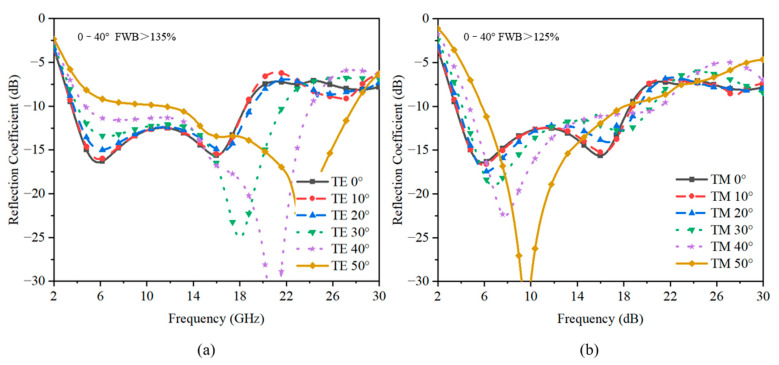
The reflection coefficient under variable incident angles. The results of the (**a**) TM polarization and (**b**) TE polarization.

The performance of the design under different oblique angles of incidence was also studied to determine the suitability of the absorber for applications demanding stable performance at large oblique angles. Figure 8a illustrates the simulated TE reflection coefficients of the absorber under oblique incidences. The results show clearly that the −10 dB reflection band extended from about 18 GHz to 26 GHz as the incident angle increased. In Figure 8b, when the incident angle increased to 40°, no significant occurrence of the reflection coefficients’ deterioration of TM polarization was observed around the frequency of operation. Overall, the absorption bandwidth varied slightly at a low frequency band and optimizes obviously at a high frequency band. So, the presented absorber has good angular stability up to about 50°.

**Figure 9:**
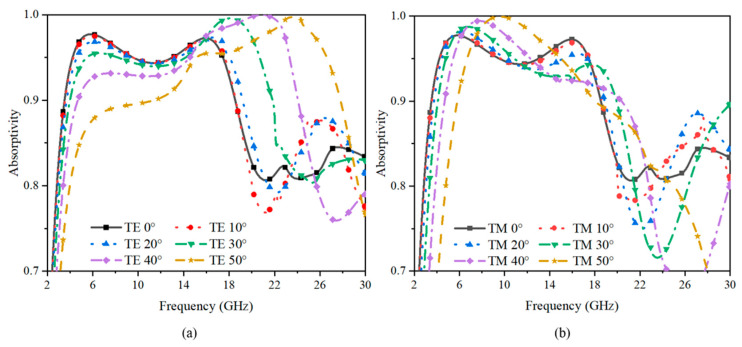
The absorptivity under variable incident angles. The results of the (**a**) TE polarization and (**b**) TM polarization.

To show more clearly the absorption performance of the presented structure, the absorptivity results are shown in Figure 9. The figure illustrates that the absorptivity could be maintained above 90% in the operating band when the incident angle increased up to 50°, which means that the structure has good absorption stability.

### 3.3. Analysis of Polarization Insensitivity

**Figure 10:**
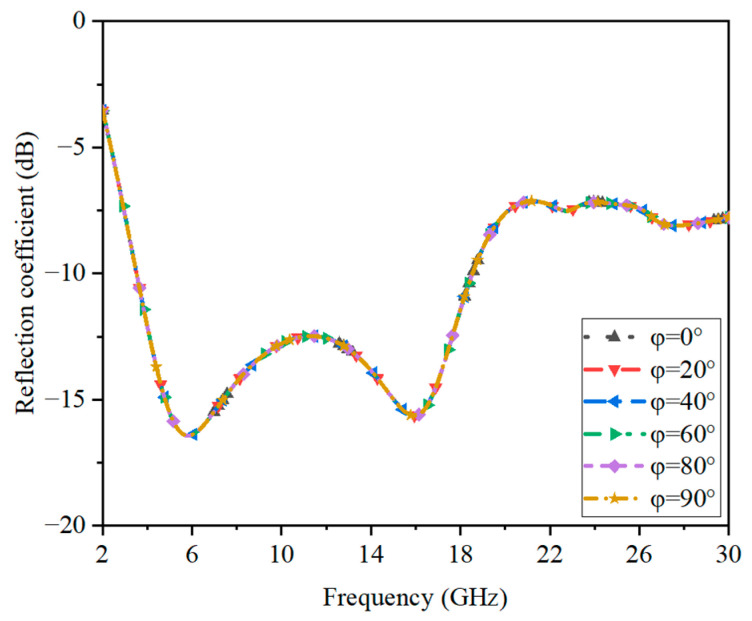
The reflection performance of the presented absorber under different polarization angles.

In Figure 10, the reflection coefficients under different polarization angles are shown to prove the presented structure had polarization insensitivity. The angle of polarization was rotated from 0° to 90°. The results had no differences, which illustrates that the absorber featured polarization-insensitive performance.

### 3.4. Analysis of the Electric Field Distributions at Normal and Oblique Incidence

**Figure 11:**
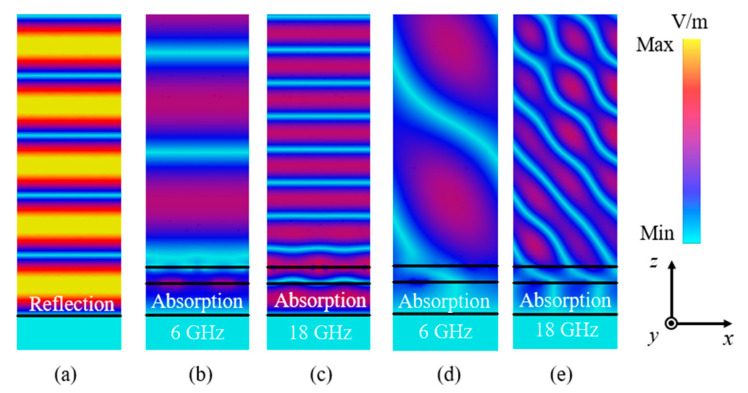
Electric field distributions on the plane of the propagation direction of the incident wave. (**a**) Reflection phenomenon of the EM waves; (**b**,**c**) absorption phenomenon at 6 GHz and 18 GHz under the normal incident angle, respectively; (**d**,**e**) absorption phenomenon at 6 GHz and 18 GHz under the 50° incident angle, respectively.

To verify the absorption performance of the proposed structure, the electric field distributions along the +*z*-axis are shown in Figure 11. It is seen in Figure 11a that the higher energy near the structure represented the generation of the standing wave phenomenon, indicating that the incident electromagnetic wave was reflected by the structure. Good absorptive behavior was achieved at the frequencies of 6 GHz and 18 GHz under the normal incident angle, with no transmission performance due to the ground plane in Figure 11b,c. It is worth pointing out that there was no standing wave generated within the two frequency bands, which indicated little energy was reflected by the presented structure, and a good stealth performance was provided. Moreover, Figure 11d,e also showed good absorption phenomena under the oblique incident angle. These phenomena were consistent with the aforementioned analysis, and the desired performances were obtained.

### 3.5. Effect of the Different Square Resistance of Graphene

**Figure 12:**
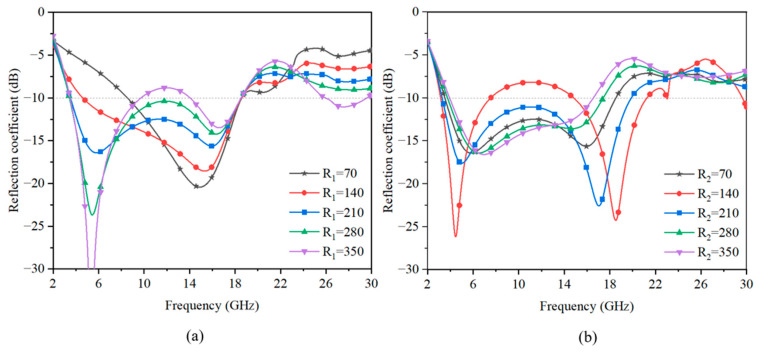
The magnitude of the reflection coefficient versus the different square resistance of the graphene structure on (**a**) lossy layer I and (**b**) lossy layer II.

Then, the sheet resistance of the graphene flake was analyzed. Figure 12a,b illuminate the reflection performance of the FSS absorber structure with different sheet resistances of *R*_1_ and *R*_2_ both in the range of 70–350 Ω/sq. As shown in Figure 12a, the *R*_1_ sheet resistance mainly affected the lower frequency absorption peak. In Figure 12b, it can be observed that the *R*_2_ sheet resistance mainly affected the absorbing bandwidth.

**Figure 13:**
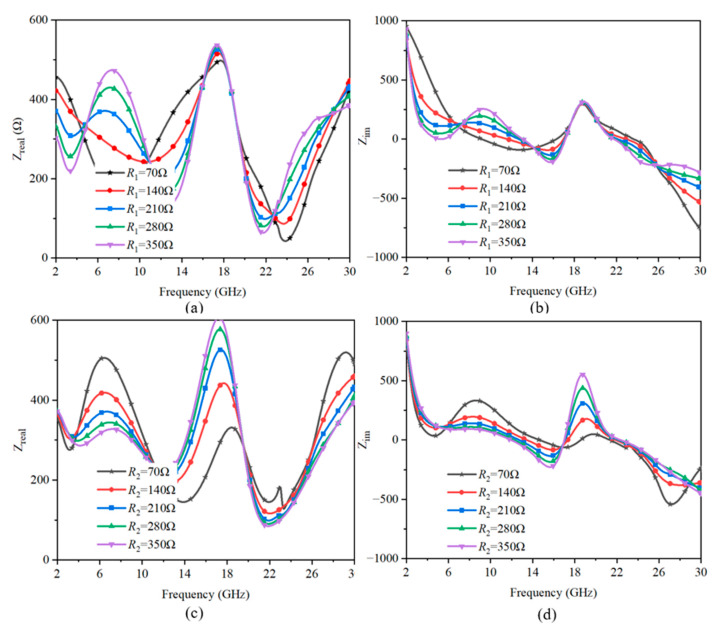
Influences of the different square resistance of graphene to the impedance. The influence of *R*_1_ on (**a**) the real part and (**b**) the imaginary part. The influence of *R*_2_ on (**c**) the real part and (**d**) the imaginary part.

The changes in the sheet resistance *R*_1_ and *R*_2_ directly influenced the real part and imaginary part of the impedance. As shown in Figure 13a,b, *R*_1_ mainly affected the real part at the low-frequency absorption band and slightly affected the high-frequency absorption band. Figure 13c,d indicate that *R*_2_ influenced the real part at both the low-frequency and high-frequency absorption peaks. Meanwhile, the frequency point near zero of the imaginary part of the impedance moved, which means the range of the absorption band may change. The analysis of the real and imaginary parts of the impedance corresponded well with the absorption performance in Figure 12.

The comparison with FSS absorbers in other references is shown in Table 3. It can be seen that the absorber in this communication has polarization insensitivity, wide reflection coefficient bandwidth below −10 dB and high angle stability.

## 4. Conclusions

In this article, a graphene-coating ultra-wideband FSS-based absorber with angular stability and polarization insensitivity performances was proposed. To mitigate the absorption performance degradation with the increasing incident angle, the optimal impedance matching effect was designed at the oblique incident angle. At the same time, graphene coating was used to obtain better broadband absorption and angular stability. As a result, the presented structure had a stable absorption performance up to 50°. It is worth pointing out that the FWB of the presented absorber maintained 136.4% up to 40°. Then, the ECM method was used to analyze the mechanism of the unit structure. According to these results, the proposed absorber may have application potential in the field of stealth.

## Figures and Tables

**Table 1:** The geometrical design parameters.

Parameter	*a*	*h*_1_	*h*_2_	*m*	*n*	*d*_1_	*d*_2_	*b*
Value (mm)	13	4	8	3.5	5	2	4	1.2

**Table 2:** The values of the parameters.

Parameter	*C*_1_	*C*_2_	*C*_3_	
Value (pF)	0.052	0.019	1.35	
Parameter	*L*_1_	*L*_2_	*L*_3_	*L*_4_
Value (nH)	1.95	1.85	0.001	0.1

**Table 3:** Performance comparison.

Ref.	Absorption Band (GHz)	FWB (%)	Angular Stability	Polarization Insensitivity	Loss Module
[3]	2.89–8.85	101.5	TE30°/TM45°	Not reported	Lumped resistors
[4]	2.69–12.04	127	TE45°/TM45°	Not reported	Lumped resistors
[18]	2.73–7.54	93.6	TE45°/TM60°	√	Graphene
[20]	3.5–13	115.67	TE30°/TM30°	√	Lumped resistors
[31]	6.7–20.58	101.7	TE45°/TM30°	√	Lumped resistors
This work	3.5–18.5	136.3	TE50°/TM50°	√	Graphene

## Data Availability

Not applicable.
